# Fungal infection detected from a peripheral blood smear review

**DOI:** 10.1515/almed-2025-0131

**Published:** 2025-10-02

**Authors:** Mireia Pallarés, Jorge Medina, Marc Garreta, Emma Padilla, Teresa Villalba, Josefa Pérez, Antonio Casabella

**Affiliations:** Department of Haematology, CATLAB, Viladecavalls, Catalunya, Spain; Department of Microbiology, CATLAB, Viladecavalls, Catalunya, Spain; Department of Microbiology, Hospital Universitari Parc Taulí, Sabadell, Catalunya, Spain; Department of Microbiology, Institut d’Investigacio i Innovacio Parc Tauli, Sabadell, Catalunya, Spain

**Keywords:** cytoplasmic inclusions, histoplasmosis, peripheral blood film

## Abstract

**Objectives:**

Histoplasmosis is a lung and blood disease caused by a dimorphic fungus, *Histoplasma capsulatum*, which causes non-specific clinical manifestations making differential diagnosis aimed at its detection difficult.

**Case presentation:**

We explore the case of a 28-year-old man whose blood test revealed pancytopenia, which led to review of the blood film, which showed yeast-like cytoplasmic inclusions in some of the neutrophils, suggestive of *H. capsulatum* infection. Given the suspicion, the laboratory expanded tests for the detection of fungal biomarkers such as galactomannan antigen and B-D-glucan, in addition to HIV serology, which were positive.

**Conclusions:**

This case highlights the importance of the morphological review of the blood film in the laboratory, as it provides very valuable and relevant information for the diagnosis of many diseases. As well as the importance of communication between the different departments in the laboratory.

## Introduction

Histoplasmosis is a lung and blood disease caused by the microorganism *Histoplasma capsulatum*, reported worldwide, but considered endemic to the Americas. *H. capsulatum* is a dimorphic fungus that at room temperature is found in a filamentous saprophytic form (mould), but at 37 °C it grows as a yeast, the latter being its infectious form. Infection occurs after inhalation of spores present in soil or dust contaminated with bird or bat droppings [1], [2], [3]. Risk factors include prolonged exposure, age over 55 years, childhood and immunosuppression in HIV patients, transplant recipients on active immunosuppressive therapy or with other immunodeficiencies [1], [2], [3].

## Case presentation

A 28-year-old man from Ecuador was admitted to his primary care center due to fever, general malaise, hyporexia, diarrhoea, and weight loss after 4 weeks of evolution. Physical examination revealed papular and pigmented skin lesions on the trunk and extremities, excluding the palms and soles of the feet. According to the patient, these lesions appeared in the last week.

The patient’s medical history includes a fracture of the calcaneus with residual limitation of ambulation and dislocation of the shoulder and right wrist. He is a snorting cocaine user and denies high-risk sexual relationships.

A complete blood count, proteinogram, basic biochemical, hepatic, renal, and hormonal profile, and serologies were requested.

The biochemical profile showed altered liver function – alanine aminotransferase 90.6 U/L (RV: 0–40.8); aspartate aminotransferase 310.2 U/L (RV: 0–40.2); gamma glutamyltransferase 511.8 U/L (RV: 10.2–71.4); alkaline phosphatase 556.8 U/L (RV: 40.2–129) − and elevated acute reactants – ferritin>160,000 ng/mL (RV: 30–400); C-reactive protein 139.66 mg/L (RV: 0–5) and erythrocyte sedimentation rate 64 mm/h (RV: 0–10).

Haematological data showed anemia with haemoglobin of 93 g/L (RV: 130–170); leukopenia of 2.75×10^9^/L (RV: 3.9–9.5); lymphopenia of 0.46×10^9^/L (RV: 1.3–3.4); and thrombocytopenia of 56×10^9^/L (RV: 149–303).

**Figure 1:**
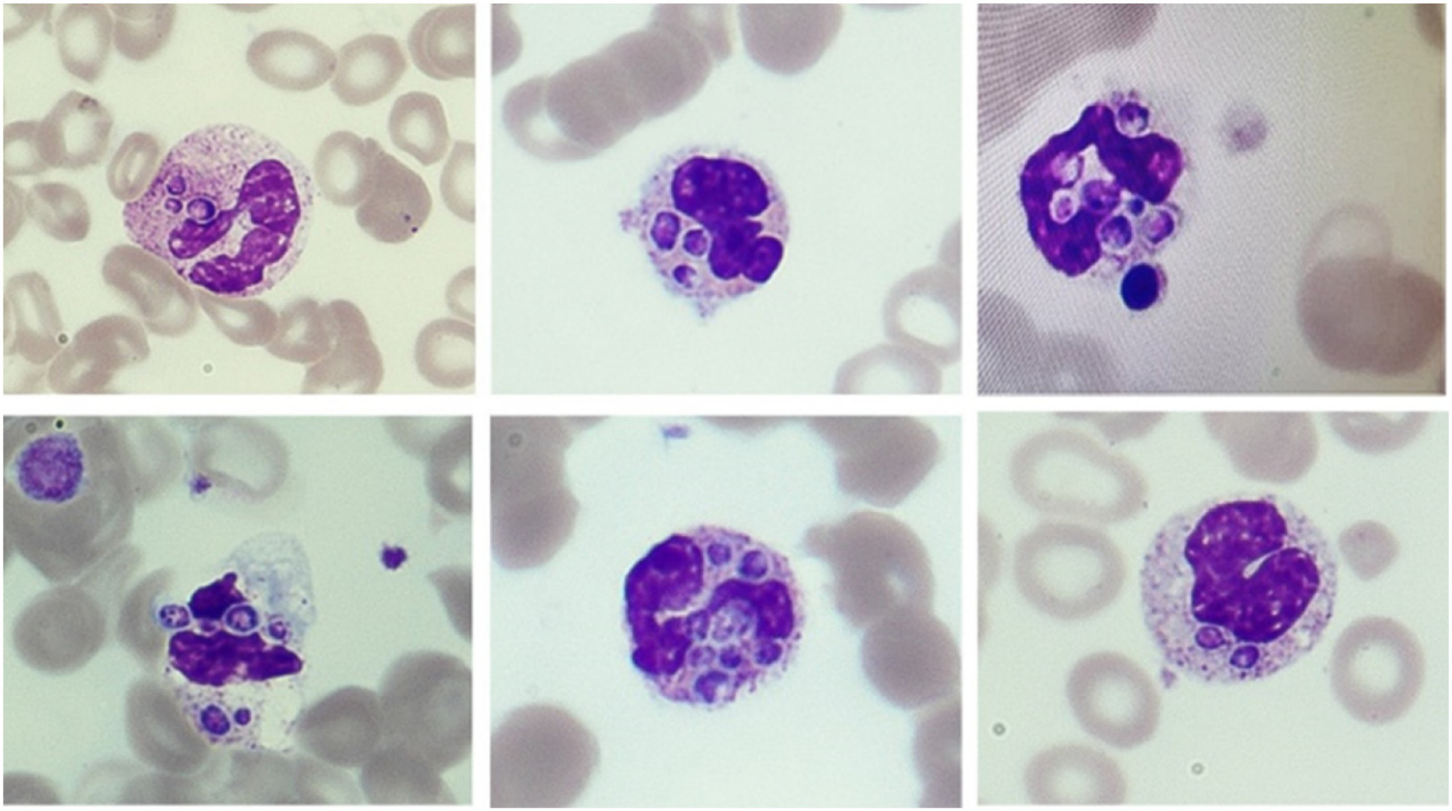
Peripheral blood film showing neutrophils with yeast-like cytoplasmic inclusions suggestive of *Histoplasma capsulatum* infection (May-Grünwald Giemsa stain, 100×).

Due to the pancytopenia, the blood film was reviewed, showing neutrophils with increased granulation, some with yeast-like cytoplasmic inclusions suggestive of *H. capsulatum* infection (Figure 1).

No antibodies to hepatitis B, hepatitis C, or *T. pallidum* were detected, but the patient tested positive for HIV with a viral load of 1,300,000 copies/mL.

Given the suspicion of *Histoplasma spp.* tests for fungal biomarkers were expanded, with positive results for galactomannan antigen and 1,3-β-D-glucan. A serum sample was sent to the external reference laboratory for a PCR testing for *H. capsulatum,* which was negative.

The results were reported to the primary care physician and the patient was referred to his referral hospital. There, bone marrow samples and a biopsy of the skin lesions were obtained for culture. Both samples were incubated at 30 °C and 37 °C and a fungus was isolated and identified as *Histoplasma spp.* by MS-TOFF mass spectrometry.

**Figure 2:**
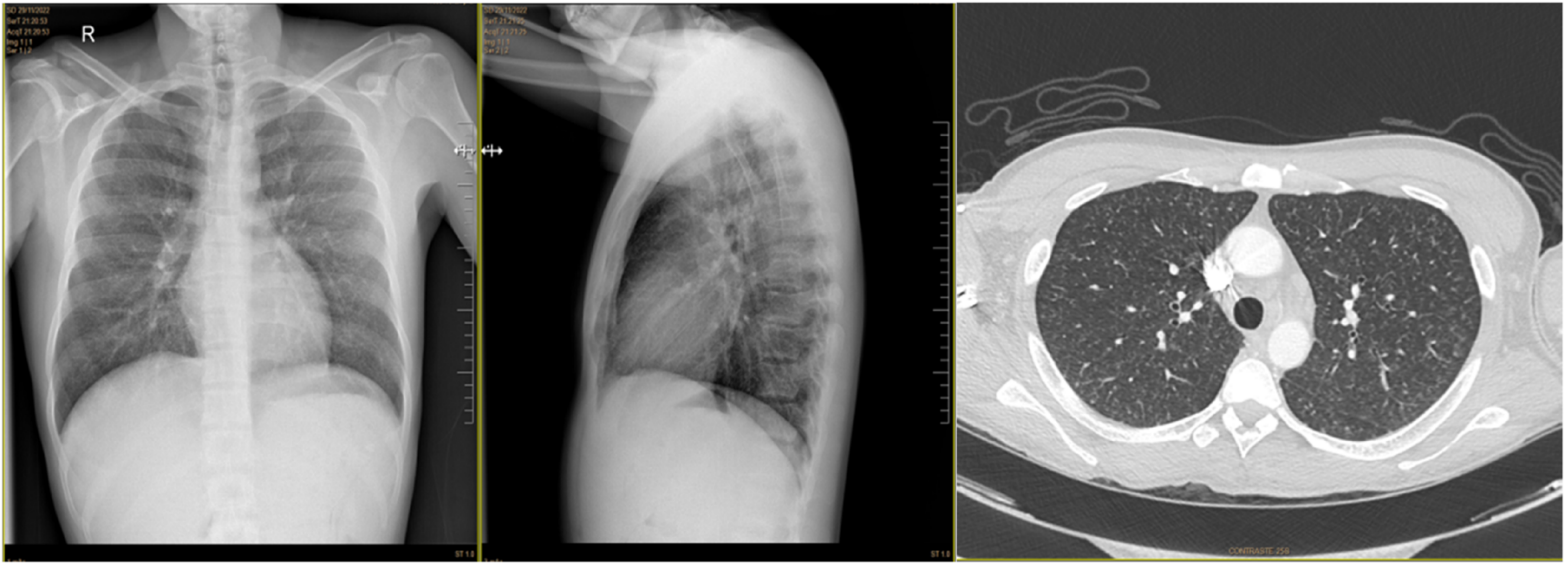
Chest computed tomography with lesions suggestive of infection.

Chest computed tomography (CT) revealed diffuse micronodular lung involvement with a miliary pattern (Figure 2). A quantiferon test was therefore performed, which was negative, thus excluding the possibility of tuberculosis infection.

In pathological anatomy, specific stains for fungi (Gomori, Giemsa, and PAS-D) were performed on the bone marrow aspirate and skin biopsy, showing forms compatible with *Histoplasma. spp.*

The patient was finally diagnosed with a disseminated *Histoplasma* infection and received standard treatment with amphotericin B followed by itraconazole with good response and improvement of symptoms.

## Case evolution

After discharge, the patient showed a favourable clinical evolution with weight and appetite gain and good tolerance to treatment, but after several months, he was poorly adherent to antiretroviral and antifungal therapy. Finally, he was lost to follow-up without the possibility of obtaining a sample to confirm negativisation.

## Discussion

The initial histoplasmosis infection develops in the lungs and usually remains localised, but in some cases, it can spread via the blood to other organs. There are several clinical presentations depending on the immunity of the patient, such as subclinical histoplasmosis; acute primary histoplasmosis with non-specific symptoms such as fever, cough, myalgia, or malaise; chronic respiratory histoplasmosis characterised by pulmonary lesions; and progressive disseminated histoplasmosis affecting various organs, such as the skin, spinal cord involvement, digestive tract, hepatosplenomegaly, lymphadenopathy, and even the central nervous system. Most cases of disseminated infection are associated with complications such as respiratory failure, gastrointestinal bleeding, disseminated intravascular coagulation, or bacterial sepsis that eventually leads to the death of the patient. [1], [2], [3], [4].

Due to the non-specific clinical features, a differential diagnosis aimed at detecting *Histoplasma spp.* is difficult to perform [1], 3].

Diagnosis is based on laboratory tests to detect the microorganism. The gold standard method is isolation by culture of various fluids (blood, sputum, bone marrow, and spinal fluid) and detection in skin, liver or spleen biopsies [1], [3], [4], [5]. In exceptional cases, as in our patient, the microorganism can be identified in the blood film [5], [6], [7].

Non-specific fungal biomarkers such as galactomannan and 1,3-β-D-glucan have a high sensitivity and PCR or detection of specific antibodies for *Histoplasma spp*. are also helpful [4], 8], 9]. The study should be completed with imaging tests to determine the degree of dissemination and the organs involved [1], 3], 5].

In our case, the microscopic finding of inclusions in neutrophils suggestive of the microorganism *H. capsulatum* helped the microbiologist to expand tests aimed at fungal detection such as B-D-glucan and galactomannan antigen.

On the other hand, the negative PCR result for *Histoplasma* was controversial, which could be explained by the fact that technique was not well optimised due to the low prevalence in our population and the difficulty of extracting DNA in fungi, [4], 5], 8], 9]. Given the findings and the clinical picture were compatible, the patient was started on treatment, which was confirmed after four weeks with the growth of a dimorphic fungus in the culture of all the samples studied.

Treatment of histoplasmosis varies according to the severity of the clinical picture and the immunity of the patient. In general, for mild or asymptomatic forms with good immunity, no treatment is required, while for moderate acute, localised, or chronic forms itraconazole is recommended. In severe disseminated forms it is recommended to start treatment with amphotericin B followed by itraconazole for at least 1 year [1], 3], 10].

## Conclusions

The review of morphology in the blood smear provides very valuable and relevant information that can be of great help in the diagnosis of haematological diseases as well as other pathologies [6], 7].

Molecular biology has allowed the introduction of new techniques used for the detection and identification of various microorganisms, such as PCR, which provides results with high levels of sensitivity and specificity. However, the sensitivity of this test varies according to the type of sample, giving less sensitive results in blood samples [4], 5], 8].

For this reason, conventional study methods such as culture remain the reference methods for the diagnosis of many microorganisms [4], 5].

Finally, this case emphasizes the importance of a good communication between the different departments of the laboratory is essential to expand the relevant studies in the shortest possible time to help guide a suspected diagnosis and thus be able to inform the clinician of the different results, together to establish an accurate final diagnosis.

## References

[1] Barros N, Wheat JL, Hage C (2023). Pulmonary histoplasmosis: a clinical update. J Fungi (Basel).

[2] Adamian CMC, Lima MMA, Martins AAF, Aragão MC, Carvalho MS, Meneses GC (2022). Progressive disseminated histoplasmosis in HIV-positive patients. Int J STD AIDS.

[3] Kauffman CA (2007). Histoplasmosis: a clinical and laboratory update. Clin Microbiol Rev.

[4] Toscanini MA, Nusblat AD, Cuestas ML (2021). Diagnosis of histoplasmosis: current status and perspectives. Appl Microbiol Biotechnol.

[5] Muñoz CO, Cano LE, González A (2010). Detección e identificación de Histoplasma capsulatum por el laboratorio: de los métodos convencionales a las pruebas moleculares. Infec.

[6] Santos Júnior CJD, Rocha TJM, Souza AKP (2022). Disseminated histoplasmosis diagnosed through blood smear. Rev Soc Bras Med Trop.

[7] Asperges E, Cavanna C, Mollaschi EMG, Seminari EM (2021). A case report of disseminated histoplasmosis in AIDS diagnosed through peripheral blood smear. Curr HIV Res.

[8] José Buitrago M, Gómez-López A, Monzón A, Rodríguez-Tudela JL, Cuenca-Estrella M (2007). Evaluación de una técnica de PCR cuantitativa para el diagnóstico clínico de la histoplasmosis importada. Enfermedades Infecciosas y Microbiología Clínica.

[9] De Lima Sampaio I, Lima Freire AK, Morishi Ogusko M, Ignez Salem J, Braga De Souza JV (2012). Selection and optimization of PCR-based methods for the detection of Histoplasma capsulatum var. capsulatum. Revista Iberoamericana de Micología.

[10] Rajme-López S, González-Lara MF, Rangel-Cordero A, Ponce-de-León A (2023). Histoplasma capsulatum prosthetic joint infection. Med Mycol Case Rep.

